# Smooth 3D Dubins Curves Based Mobile Data Gathering in Sparse Underwater Sensor Networks

**DOI:** 10.3390/s18072105

**Published:** 2018-06-30

**Authors:** Wenyu Cai, Meiyan Zhang

**Affiliations:** 1 School of Electronics and Information, Hangzhou Dianzi University, Hangzhou 310018, China; 2 School of Electrical Engineering, Zhejiang University of Water Resources and Electric Power, Hangzhou 310018, China

**Keywords:** underwater sensor networks, mobile data gathering, autonomous underwater vehicles, 3D Dubins curve

## Abstract

Sensory data collection is one of the most important concerns in underwater sensor networks (USNs). Because full connectivity cannot be guaranteed, mobile data gathering with autonomous underwater vehicles (AUVs) is widely used in sparse three-dimensional (3D) USNs to solve energy-imbalance problems between different sensor nodes. AUVs with relatively abundant energy and storage can collect sensory data from one sensor node to transmit to another node, so as to avoid energy-intensive multi-hop transmission. As a result, movement control strategy and data collecting path planning for AUVs are very crucial for the performance of data acquisition. This paper proposes a smooth 3D Dubins curves based mobile data gathering mechanism to overcome the kinematic nonholonomic constraints of AUVs. The objective of our proposed method is to collect sensory data along smooth 3D Dubins paths, which are interpolated by continuous Bezier curves in the *Z*-axis from 2D Dubins curves. Extensive simulation results verify that the proposed method has a more efficient performance in terms of path smoothness and energy consumption; thus it is very suitable for mobile data collection in 3D underwater sensor networks.

## 1. Introduction

As a result of recent advances in underwater sensing, acoustic communication, and battery technologies, underwater sensor networks (USNs) have obtained considerable attention for deployment in diverse areas such as marine environment monitoring and underwater resource exploration [1]. Nevertheless, the limited bandwidth and energy-constrained characteristics of acoustic communication make it challenging to collect data in USNs. Moreover, the sparsity feature of USNs makes it hard to guarantee their entire connectivity. Additionally, the traditional multi-hop transmission of terrestrial sensor networks will lead to energy holes around the stationary sink, as underwater sensors closer to the sink deplete their energy faster, so as to cause an energy-consumption unbalance problem. To deal with disconnected routing and energy imbalances, numerous research works have adopted mobile data collection technology. For traditional wireless sensor networks, an energy-aware path-construction (EAPC) algorithm that selects an appropriate set of data collection points and constructs a data collection path was investigated in [2], two approaches including data collection using a data mule (MULE) and sensor networks with a mobile access point (SENMA) are proposed in [3], and a computationally efficient motion-planning approach to solve the joint path planning and clustering problem by using space-filling curves is proposed in [4]. Additionally, Yang et al. [5] focus on low-delay and high-throughput opportunistic data collection in wireless sensor networks with mobile sinks (WSN-MSs) of general network topologies and arbitrary numbers of mobile sinks. These methods complete the data collection task of wireless sensor networks depending on either multi-hop routing or mobile sinks. For limited acoustic communication in USNs, whole network connectivity cannot be guaranteed all of the time, such that multi-hop-routing-based data collection methods do not perform well.

Currently, a widely used data collection method for USNs is by the assistance of one autonomous underwater vehicle (AUV) or multiple AUVs [6]. Usually AUVs with sufficient energy are employed to collect sensory data in sparse three-dimensional (3D) USNs. Generally speaking, mobile sinks can visit underwater sensors and collect data from them while moving along a specific path, thus avoiding the long-distance energy-intensive transmission of sensory data [7,8]. As a result, AUV-assisted USNs can efficiently improve data collection and extend the survival time of underwater sensors. Inspired by this, some energy-efficient data collection algorithms were proposed to balance the energy consumption between underwater sensor nodes. An enhanced energy-balanced data-transmission protocol was proposed to overcome energy inefficiency [9]. Also of relevance, to improve the effectiveness of mobile data collection, the mobility influence of AUVs was investigated by Janardanan and Jacob [10]. A multi-hop data-gathering scheme was provided by [11] to forward data to the designated sink. An underwater acoustic sensor network with one mobile surface node to collect data from multiple underwater nodes was investigated in [12], whereby the mobile destination requests retransmission from each underwater node individually by employing the traditional automatic-repeat-request (ARQ) protocol. Zhang et al. [13] aimed to overcome the shortcomings of the wolf-pack algorithm (WPA) and improve three intelligent behaviors of the WPA, namely, scouting, summoning, and beleaguering. As a survey paper, Zeng et al. [14] studied path planning for the persistent autonomy of autonomous underwater vehicles. Han et al. [15] proposed a probabilistic neighborhood location-point covering-set-based data collection algorithm with obstacle avoidance for underwater acoustic sensor networks. However, most of this research was focused on the two-dimensional (2D) mobile data gathering (MDG) problem. It is noted that the efficiency and smoothness of the planned paths for mobile data collection can be improved [14].

Previous research on MDG using AUVs has focused primarily on a reduction in the length of the AUVs’ traveling paths. However, as a result of well-known kinematic constraints, the data collecting trajectory of AUVs should be of geometric continuity and smoothness [16]. The Dubins based traveling salesman problem (DTSP) [17] is designed to generate the minimum total length while visiting a set of sensor nodes with Dubins curves, but it can only be used in 2D planes. Cao et al. [18] propose a linear interpolation method to convert 2D Dubins curves to 3D Dubins curves for underwater glider path planning. However, linear interpolation in the *Z*-axis fails to satisfy the 3D smoothness constraint. Kuniaki et al. propose a smooth path generation method for automated vehicles on the basis of the Bezier curve [19], but it is only applicable to 2D planes. Although these algorithms can provide optimal or near-optimal paths that pass through all predefined waypoints, they cannot guarantee smoothness in 3D regions. To deal with these problems, given a mobile AUV and a set of underwater sensors, this paper proposes continuous and smooth 3D Dubins curves with Bezier interpolation in the *Z*-axis for MDG of USNs.

*Statement of Contributions*: (1) This paper extends the traditional 2D Dubins curve to a 3D Dubins curve with continuous Bezier interpolation instead of the linear interpolation method; (2) this paper also designs the interpolation of smooth 3D Bezier curves in the *Z*-axis and proves its smoothness. Although the curvature–continuous-path planning algorithm has been used in unmanned aerial vehicles (UAVs) [20], it has not yet been well studied for USNs. Moreover, the Catmull–Rom curve construction method to satisfy the curvature continuity condition in [20] is overladen for USNs. To the best of our knowledge, this is the first report to design smooth 3D curves for MDG of USNs. Extensive results verify that the proposed method can achieve trajectory smoothness with little extra cost.

The rest of this paper is organized as follows. Section 2 presents the problem statement and network model. In Section 3, smooth 3D Dubins curves with the Bezier interpolation based MDG algorithm are presented. Simulation results are shown in Section 4, followed by the conclusions in Section 5.

## 2. Preliminaries and Problem Statement

**Figure 1:**
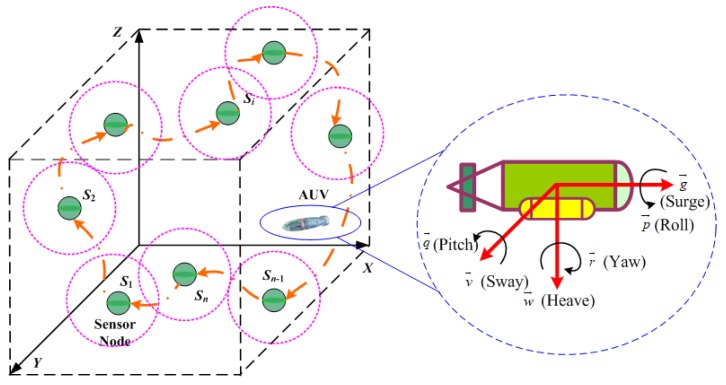
Network topology and dynamics of autonomous underwater vehicles (AUVs) (*n* = 10 sensor nodes and one AUV deployed randomly in a three-dimensional underwater region).

As depicted in Figure 1, there are *n* sensor nodes $\left(S_{i},i=1,2,\cdots ,n\right)$ with coordinates $\left(x_{i},y_{i},z_{i}\right)\in R^{3}$ and one mobile AUV deployed randomly in a W×W×W cubic underwater space. Each underwater sensor node has the ability of monitoring parameters of the surroundings and transmitting data via acoustic communication. Compared with the vast monitoring region of USNs, the drifting movement of underwater sensors by oceanic current can be ignored. Additionally, it is assumed that the battery energy of mobile AUVs is enough to support the complete traversal of a scheduled trajectory. As USNs might be deployed sparsely, sensory data gathering in the manner of traditional multi-hop transmission between underwater sensors is not applicable. The investigated USN model considers the larger-range 3D topological property but ignores such factors as aquatic medium and acoustic communication.

The nonholonomic motion constraint requires that AUVs move along a continuous and smooth path to satisfy kinematic constraints. As shown in Figure 1, the kinematics of AUVs with spatial position $\left[x_{A},y_{A},z_{A},\psi_{A}\right]$ are defined as

(1) $$\left\{\begin{array}{c} x_{A}\left(\tau +1\right)=x_{A}\left(\tau \right)+\rho \times \left(\vec{g}cos\psi_{A}-\vec{v}sin\psi_{A}\right),\\ y_{A}\left(\tau +1\right)=y_{A}\left(\tau \right)+\rho \times \left(\vec{g}sin\psi_{A}+\vec{v}cos\psi_{A}\right),\\ z_{A}\left(\tau +1\right)=z_{A}\left(\tau \right)+\rho \times \vec{\omega },\\ \psi_{A}\left(\tau +1\right)=\psi_{A}\left(\tau \right)+\rho \times \vec{\gamma }, \end{array}\right.$$

where $x_{A}$, $y_{A}$, and $z_{A}$ are the positions in the north, east, and downward axes, respectively; $\psi_{A}$ denotes the rotation angle around the downward axes; and ρ is a scalar governing the trajectory updating of AUVs, that is, the time interval for updating the kinematics model. Additionally, $\vec{g}$, $\vec{v}$, and $\vec{\omega }$ are the linear velocities in surge, sway, and heave and $\vec{p}$, $\vec{q}$, and $\vec{\gamma }$ are the angle velocities in roll, pitch and yaw, respectively [21].

The smoothness of curves and junctions can be measured by geometric and parametric continuity. First of all, we propose the definition of $G^{1}$ continuous curves:

*$G^{1}$ continuous curves*: Let $P(s_{0},s_{1}:s)$ and $Q(t_{0},t_{1}:t)$ be two parametric curves $P\left(s_{1}\right)=Q\left(t_{0}\right)=J$ and $\dot{P}\left(s\right){{|}_{s_{1}}=\dot{Q}\left(t\right)|}_{t_{0}}$; then the two parametric curves meet at joint *J* with $G^{1}$ continuity. If $\dot{P}{\left(s\right)|}_{s}$ and $\dot{Q}{\left(t\right)|}_{t}$ are both continuous, the connecting curves by P(s) and Q(t) belong to $G^{1}$ continuous curves.

With this, we can define and design smooth curves in 3D space. The investigated 3D smooth curves are defined as follows:

*3D smooth curves*: Let P(s)=[x(s),y(s),z(s)] and Q(t)=[x(t),y(t),z(t)] be two regular parametric 3D curves in a 3D region, where s∈[0,1] and t∈[0,1] are the parameters with 0 and 1 denoting the starting and ending points, respectively. Moreover, P(s) and Q(t) are $G^{1}$ continuous curves in both the *X*–*Y* plane and *Z*-axis. If P(1)=Q(0)=J and $\dot{P}\left(s\right){{|}_{s=1}=\dot{Q}\left(t\right)|}_{t=0}$, then the two parametric curves meet at joint *J* with $3D-G^{1}$ continuity; that is, these curves belong to 3D smooth curves.

In other words, 3D smooth ($3D-G^{1}$ continuous) curves refer to such curves satisfying $G^{1}$ continuity in the *X*-, *Y*-, and *Z*-axes.

In this paper, the sensory data gathering process is involved in the cruising process of AUVs; that is to say, the AUV does not have to stop at a certain collection position to receive data. From this perspective, 3D path planning for MDG aims to determine a smooth path from an initial node to a final node through a complicated 3D underwater space. The investigated 3D-smooth-curves-based MDG in this paper collects sensory data from all underwater sensors one by one with predefined 3D smooth curves.

We let $L_{ij}=\int_{0}^{1}\left|C_{ij}\left(s\right)\right|ds$ be the length of 3D smooth curve $C_{ij}\left(s\right)\left(i\neq j\right)$; $P(L_{ij})$ and $E_{i}$ denote the energy consumption of cruising along smooth curve $L_{ij}$ and the data collecting approaching the sensor node $S_{i}$, respectively. The indicator $k_{ij}=1$ denotes that the AUV is assigned to pass sensor node $S_{j}$ as the the next sensor node after $S_{i}$, and otherwise $k_{ij}=0$. Therefore, the optimization objective of the 3D smooth curves planning-based MDG is given as

(2) $$\begin{array}{cc} \mathrm{minimize}\;\;\; & P\left(k\right)=\sum_{i=1}^{n}\sum_{j=1}^{n}k_{ij}\cdot P\left(L_{ij}\right)+\sum_{i=1}^{n}E_{i}, \end{array}$$

(3) $$\begin{array}{cc} \mathrm{subject}\;\mathrm{to}\;\;\; & \sum_{i=1}^{n}k_{ij}=1,\;\;\;\forall j=1,2,\cdots ,n, \end{array}$$

(4) $$\begin{array}{c} \sum_{j=1}^{n}k_{ij}=1,\;\;\;\forall i=1,2,\cdots ,n, \end{array}$$

(5) $$\begin{array}{c} C_{ij}\in 3D-Smooth-Curves,\forall i,j=1,2,\cdots ,n, \end{array}$$

where Equation (2) is our objective function representing the total energy consumption in collecting all sensory data. For the sake of simplicity, $E_{i}$ is neglected in the following section, as it is much smaller than the cruising energy consumption. Equations (3) and (4) guarantee that all underwater sensors are visited by the AUV exactly once. The novel constraint in Equation (5) is our focusing point of this paper. The above problem can be modeled as a type of a new DTSP and can be solved by a certain heuristic method. As far as a 3D smooth trajectory is concerned, the AUV can perform the data collection task using shorter-range acoustic communication to reduce the energy consumption in a cruising manner.

## 3. Smooth 3D Dubins Curves Planning-Based Mobile Data Gathering Algorithm

It has been mentioned above that mobile AUVs have the ability of passing through all predefined underwater sensors along a smooth trajectory to collect sensory data in the desired mission region. In this section, we detail our Bezier interpolation based 3D Dubins curves construction method for mobile data collection in USNs.

### 3.1. 3D Dubins Curves with Bezier Interpolation

**Figure 2:**
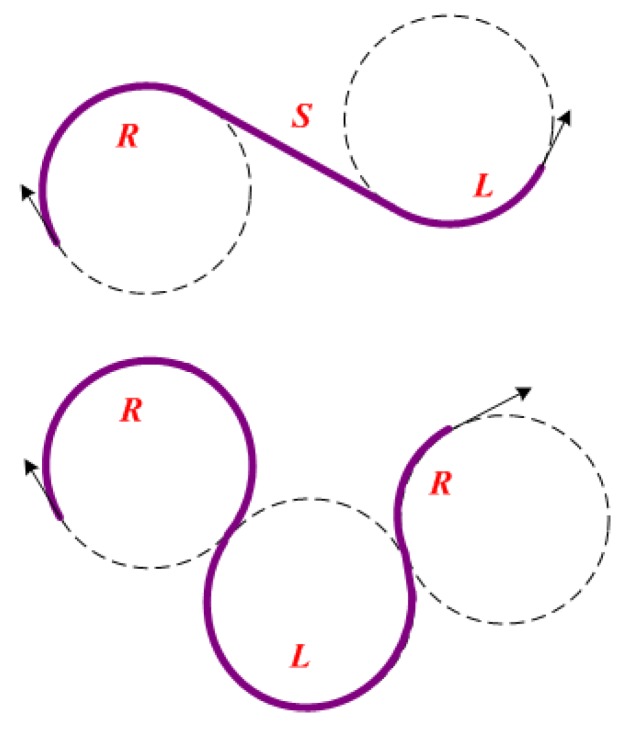
Two cases of Dubins curves.

**Figure 3:**
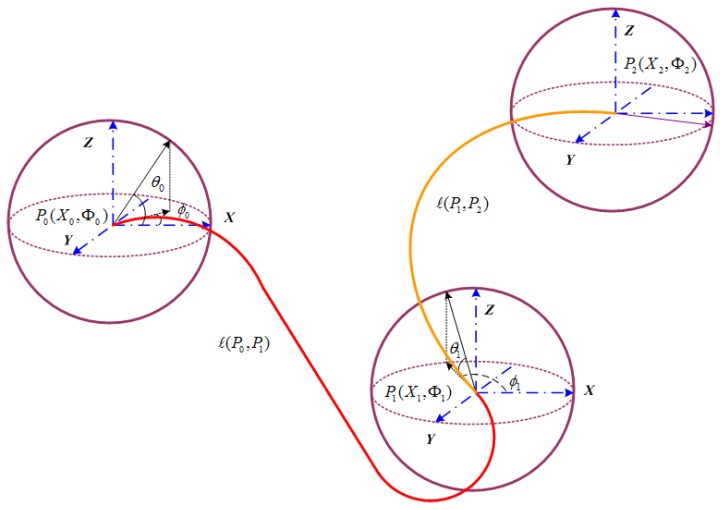
Principle of three-dimensional (3D) Dubins curves.

Dubins curves of the two cases in Figure 2 have been proven to realize smooth paths in 2D planes [17]. The Dubins curves satisfy the motion constraints by a combination of maximum curvature arcs (*R* or *L*) or a straight line segment (*S*). The presence of kinematic constraints of AUVs implies that the distance between pairs of underwater nodes depends on the incoming and outgoing directions of sensor nodes. The incoming and outgoing heading angles are selected on the basis of the optimal type of Dubins curve, which is one of {RSL,RSR,LSL,LSR,RLR,LRL}. Our former works [22,23] extend 2D Dubins curves to 3D Dubins curves from sensor node $S_{i}\left(x_{i},y_{i},z_{i},\Phi_{i}\right)$ to sensor node $S_{j}\left(x_{j},y_{j},z_{j},\Phi_{j}\right)$ (as in Figure 3) by assigning each $\left(x,y,\phi \right)\in R^{2}$ and $\left(x,y,z,\Phi \right)\in R^{3}$ with

(6) $$\begin{array}{c} z=z_{i}+\frac{L_{\left(x_{i},y_{i}\right)}^{\left(x,y\right)}}{L_{\left(x_{i},y_{i}\right)}^{\left(x_{j},y_{j}\right)}}\times \left(z_{j}-z_{i}\right), \end{array}$$

where $L_{\left(x_{i},y_{i}\right)}^{\left(x,y\right)}$ and $L_{\left(x_{i},y_{i}\right)}^{\left(x_{j},y_{j}\right)}$ denote lengths of 2D Dubins curves from $\left(x_{i},y_{i},\phi_{i}\right)$ to (x,y,ϕ) and $\left(x_{j},y_{j},\phi_{j}\right)$, respectively.

Thus, the length of a 3D Dubins curve with linear interpolation from underwater sensor $S_{i}\left(x_{i},y_{i},z_{i},\Phi_{i}\right)$ to $S_{j}\left(x_{j},y_{j},z_{j},\Phi_{j}\right)$ is demonstrated as

(7) $$\begin{array}{c} L_{Linear-3D}=\sqrt{{\left(L_{\left(x_{i},y_{i}\right)}^{\left(x_{j},y_{j}\right)}\right)}^{2}+{\left(z_{j}-z_{i}\right)}^{2}}. \end{array}$$

**Figure 4:**
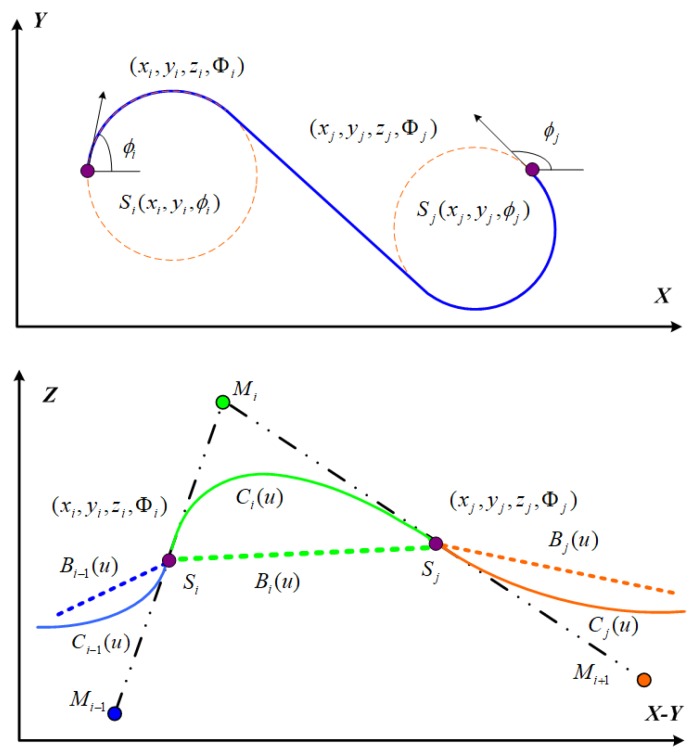
Two-dimensional (2D) and three-dimensional (3D) Dubins curves.

However, the above 3D linear interpolation fails to maintain the $G^{1}$ continuity in the *Z*-axis. Therefore, we propose Bezier interpolation based smooth 3D Dubins path planning for MDG in USNs. In the upper part of Figure 4, we use a traditional Dubins curve to design the path in the *X*–*Y* plane. In the lower part of Figure 4, we extend the 2D Dubins curve to a 3D Dubins curve from sensor node $S_{i}\left(x_{i},y_{i},z_{i},\Phi_{i}\right)$ to $S_{j}\left(x_{j},y_{j},z_{j},\Phi_{j}\right)$ by assigning *z* with a quadratic Bezier curve $C_{i}\left(u\right)$ [24]:

(8) $$z={\left(1-u\right)}^{2}z_{i}+2u\left(1-u\right)M_{i}+u^{2}z_{j},$$

where $M_{i}$ is the *Z*-axis coordinate value of a control point in the quadratic Bezier curve.

The traditional Bezier curve is defined as

(9) $$\begin{array}{c} C\left(u\right)=\sum_{t=0}^{m}B_{m,t}\left(u\right)D_{t}, \end{array}$$

where $D_{t}$ denotes end points and $B_{m,t}\left(u\right)=\frac{m\;}{t\;(m-t)\;}u^{t}{\left(1-u\right)}^{m-t}$.

For simpler quadratic Bezier curves, we have

(10) $$\begin{array}{c} C\left(u\right)={\left(1-u\right)}^{2}D_{0}+2u\left(1-u\right)D_{1}+u^{2}D_{2}, \end{array}$$

where $D_{0}$ and $D_{2}$ denote end points and $D_{1}$ denotes a control point. It is noted that the first-order derivative of a quadratic Bezier curve is continuous [24].

The approximate length of a quadratic Bezier curve can be calculated as follows [24]:

(11) $$\begin{array}{c} \begin{array}{cc} L_{z_{i}\;z_{j}} & \approx \frac{2}{3}\times \left|z_{i},z_{j}\right|+\frac{1}{3}\times \left(\left|z_{i},M_{i}\right|+\left|M_{i},z_{j}\right|\right)\\ & \geq \frac{2}{3}\times \left|z_{i},z_{j}\right|+\frac{1}{3}\times \left|z_{i},z_{j}\right|\\ & \geq \left|z_{i},z_{j}\right|. \end{array} \end{array}$$

It can be proved from the above equation that the Bezier curve interpolation method will lead to a slightly longer length than linear interpolation in the *Z*-axis. As a result, the length of a 3D Dubins curve with Bezier interpolation is

(12) $$\begin{array}{c} L_{Bezier-3D}=\sqrt{{\left(L_{\left(x_{i},y_{i}\right)}^{\left(x_{j},y_{j}\right)}\right)}^{2}+{\left(L_{z_{i}\;z_{j}}\right)}^{2}}. \end{array}$$

### 3.2. 3D Smooth Dubins Based Trajectory Construction

Although each Bezier curve between sequential underwater sensors is smooth, how can all the Bezier curves connecting all sensor nodes be smooth? The smooth property of the curves’ junction can be checked and designed by geometric continuity. This subsection presents how to maintain global smoothness for a sequence of Bezier curves. For the purpose of making explanations simpler, some variables, such as $M_{i}$ and $S_{j}$, in formulas are also considered to represent their coordinates of control nodes $M_{i}$ and sensor nodes $S_{j}$, respectively.

Each designed underwater node sequence can be mapped to a continuous Bezier curve:

(13) $$\left\{\begin{array}{c} C_{1\;2}\left(u\right)={\left(1-u\right)}^{2}S_{1}+2u\left(1-u\right)M_{1}+u^{2}S_{2},\\ C_{2\;3}\left(u\right)={\left(1-u\right)}^{2}S_{2}+2u\left(1-u\right)M_{2}+u^{2}S_{3},\\ \ldots \;\ldots \;\ldots \;\ldots ,\\ C_{n-1\;n}\left(u\right)={\left(1-u\right)}^{2}S_{n-1}+2u\left(1-u\right)M_{n-1}+u^{2}S_{n},\\ C_{n\;1}\left(u\right)={\left(1-u\right)}^{2}S_{n}+2u\left(1-u\right)M_{n}+u^{2}S_{1}. \end{array}\right.$$

To satisfy the continuous first-order derivative at junction node $S_{j}$ in Figure 4, that is, $\dot{C_{i}}\left(u\right){{|}_{u=1}=\dot{C_{j}}\left(u\right)|}_{u=0}$, where $\dot{C_{i}}{\left(u\right)|}_{u=1}=2\times \left(S_{j}-M_{i}\right)$, $\dot{C_{j}}{\left(u\right)|}_{u=0}=2\times \left(M_{i+1}-S_{j}\right)$, therefore

(14) $$M_{i}+M_{i+1}=2\times S_{j},$$

where $M_{i}$ denotes the control point between sensor nodes $S_{i}$ and $S_{j}$.

Specific to smooth-trajectory-based mobile data collection of USNs, the expected control points $M_{i}$ (i=1,2,…,n) must satisfy such equation set to guarantee that the whole trajectory is smooth, which is done using multiple Bezier curves.

(15) $$\left\{\begin{array}{c} M_{1}+M_{2}=2\times S_{2},\\ M_{2}+M_{3}=2\times S_{3},\\ \ldots \;\ldots \;\ldots ,\\ M_{n-1}+M_{n}=2\times S_{n},\\ M_{n}+M_{1}=2\times S_{1}. \end{array}\right.$$

As a result, these equations can be rewritten more clearly and concisely with a matrix equation, AM=S, where $M={\left[M_{1},M_{2},\ldots ,M_{n-1},M_{n}\right]}^{T}$, $S={\left[2S_{2},2S_{3},\ldots ,2S_{n},2S_{1}\right]}^{T}$, and

(16) $$A={\left[\begin{array}{ccccc} 1 & 1 & \ldots & 0 & 0\\ 0 & 1 & \ldots & 0 & 0\\ \ldots & \ldots & \ldots & \ldots & \ldots \\ 0 & 0 & \ldots & 1 & 1\\ 1 & 0 & \ldots & 0 & 1 \end{array}\right]}_{n\times n}.$$

The determinant and rank of matrix A can be calculated as follows:

(17) $$\left|A\right|=\left\{\begin{array}{c} 0,\;if\;n\;is\;even,\\ 2,\;if\;n\;is\;odd. \end{array}\right.$$

(18) $$Rank\left(A\right)=\left\{\begin{array}{c} n-1,\;if\;n\;is\;even,\\ n,\;\;if\;n\;is\;odd. \end{array}\right.$$

Clearly, $M=A^{-1}S$ if *n* is odd. Otherwise, M is unsolvable if *n* is even. Therefore, Bezier interpolation curves in the *Z*-axis can be determined with such a solution set. In the case of an even number of underwater sensors, we can add one virtual sensor node to the original network topology. That is to say, only the final Bezier curve in the trajectory sequence has to be designed specifically. Consequently, the interpolated 3D Dubins paths will be continuous and smooth at each sensor node and meet the requirements of kinematic constrains.

### 3.3. 3D Dubins Based Mobile Data Gathering Procedures

All in all, the proposed Bezier interpolation 3D Dubins curves based MDG algorithm has the following key steps.

Step 1: For the sake of simplicity, the smooth 3D Dubins problem with Bezier interpolation is utilized with the Euclidean distance $d_{ij}$ between USNs $S_{i}$ and $S_{j}$, which is defined as

(19) $$d_{ij}=\sqrt{{\left(x_{i}-x_{j}\right)}^{2}+{\left(y_{i}-y_{j}\right)}^{2}+{\left(z_{i}-z_{j}\right)}^{2}},$$

where i,j=1,2,…,n and i≠j.

It is noted that the visiting sequence assignment for underwater sensors is modeled as the traveling salesman problem with the following integer programming formulation:

(20) $$\begin{array}{cc} \mathrm{minimize}\;\;\; & L\left(k\right)=\sum_{i=1}^{n}\sum_{j=1}^{n}k_{ij}\cdot d_{ij}, \end{array}$$

(21) $$\begin{array}{c} \sum_{i=1}^{n}k_{ij}=1,\;\;\;\forall j=1,2,\cdots ,n, \end{array}$$

(22) $$\begin{array}{c} \sum_{j=1}^{n}k_{ij}=1,\;\;\;\forall i=1,2,\cdots ,n, \end{array}$$

where Equation (20) is our objective function representing the total distance L(k) to visit all underwater sensors. Equations (21) and (22) guarantee that all underwater sensors are visited by the AUV exactly once.

**Figure 5:**
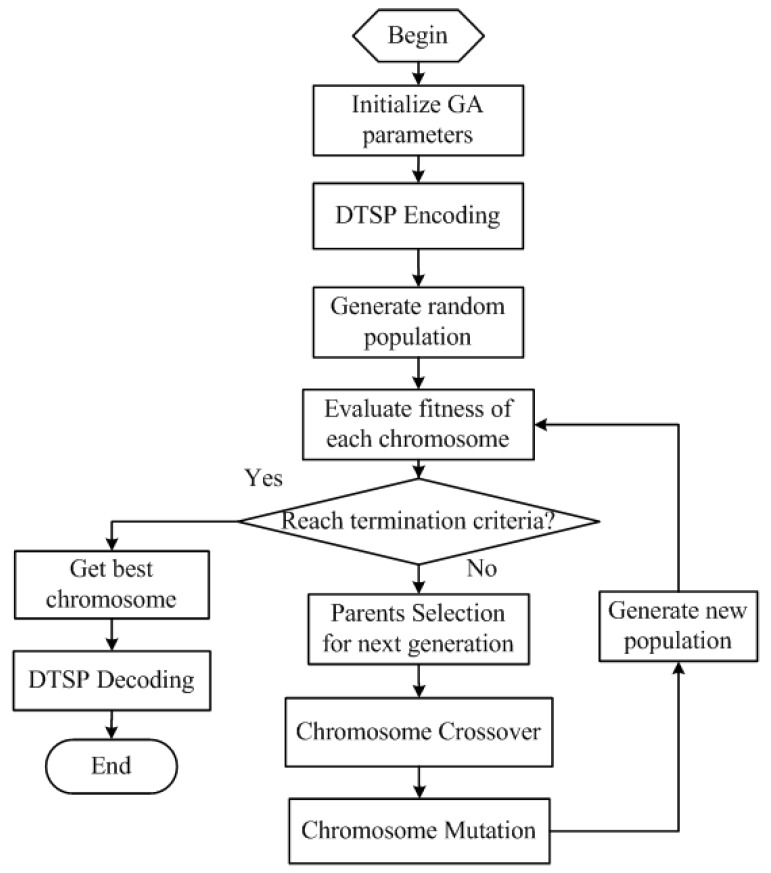
Genetic algorithm (GA)-based iteration process.

Step 2: The genetic algorithm (GA)-based heuristic algorithm [25] is used to obtain the optimal sensor node sequence. The procedures of the GA-based iteration calculation for solving the traveling salesman problem are described in Figure 5 with the following critical steps:

(a) Generate the first random population and evaluate the fitness value; the fitness function is defined as the reciprocal of Euclidean distances.
(b) Select the best solutions as parents and use chromosome crossover and mutation operations to generate a new population.
(c) Calculate the fitness value of the new population and find the best chromosome.
(d) If the best solution satisfies the terminal criteria, we obtain the best chromosome. If not, we need to repeat the above step until the best solution meets the terminal condition.

**Figure 6:**
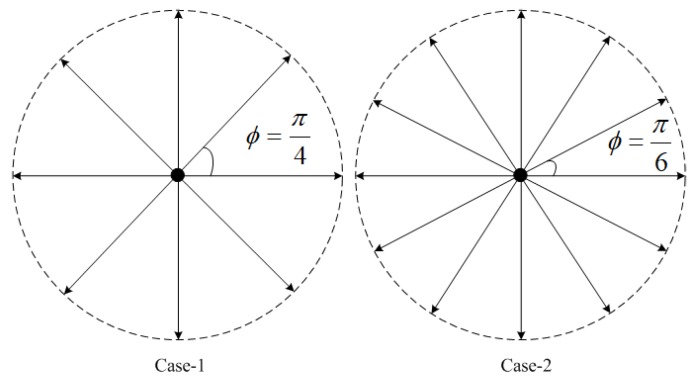
Simplified azimuth heading sets.

Step 3: The optimal orientation of azimuth headings can be selected using the decision table in [26]. For underwater sensors in the middle of the node sequence, the outgoing angle has to be the same as the incoming angle. Furthermore, simplified azimuth headings of AUVs with a finite number of directions, as in Figure 6, are confined by the following equations (cases 1 and 2) to reduce the computational complexity:

(23) $$\begin{array}{c} \mathrm{Case}\;1:\;\Phi =\{\phi =\frac{\lambda \pi }{4}|\lambda =0,1,2,\ldots ,7\} \end{array}$$

(24) $$\begin{array}{c} \mathrm{Case}\;2:\;\Phi =\{\phi =\frac{\lambda \pi }{6}|\lambda =0,1,2,\ldots ,11\}. \end{array}$$

Step 4: The optimal sequence of sensor nodes is mapped to 3D Dubins curves by 2D Dubins curves’ construction in the *X*–*Y* plane and Bezier interpolation in the *Z*-axis.

**Figure 7:**
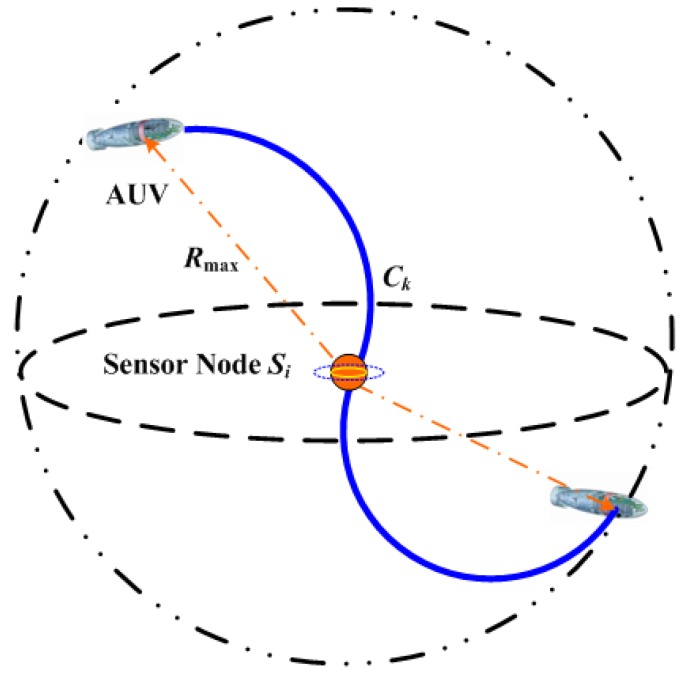
Data gathering at sensor node $S_{i}$ sphere.

Step 5: The derived smooth curves are assigned to the AUV for trajectory cruising and data gathering. In fact, it needs to be explained that the AUV should only cruise and reach within the communication range of the exact sensor’s position, as depicted in Figure 7. The detailed interactive process and protocol between sensor nodes and the AUV are omitted because of their simplicity.

As depicted in Figure 7, the communication range of an underwater sensor is $R_{max}$. We suppose there are *W* bits of sensory data to delivery and that the acoustic communication speed is *V* bit/s; $T_{C_{k}}$ denotes the cruising time across the communication sphere once the cruising speed of the AUV is normalized. To accomplish the data collecting task for underwater sensor $S_{i}$, this should be

(25) $$\begin{array}{c} T_{C_{k}}\geq \frac{W}{V}. \end{array}$$

To be more specific, the sensory data collection process $T_{C_{k}}$ should be accomplished in the specified sphere with radius $R_{max}$. The greater the communication radius, the longer the data acquisition time. However, this article assumes that the communication range is enough for data collection of the AUV. Moreover, this paper simplifies some possible influences caused by wireless acoustic communication.

## 4. Simulation Results

**Figure 8:**
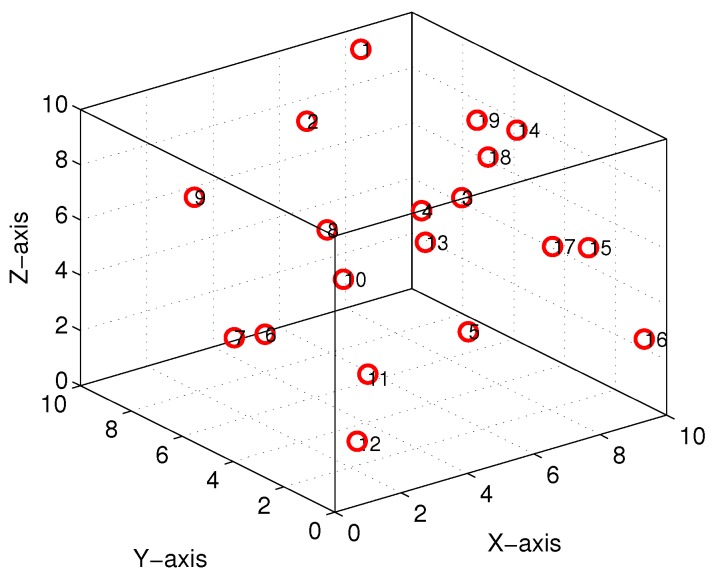
Simulation topology snapshot (n=19).

**Figure 9:**
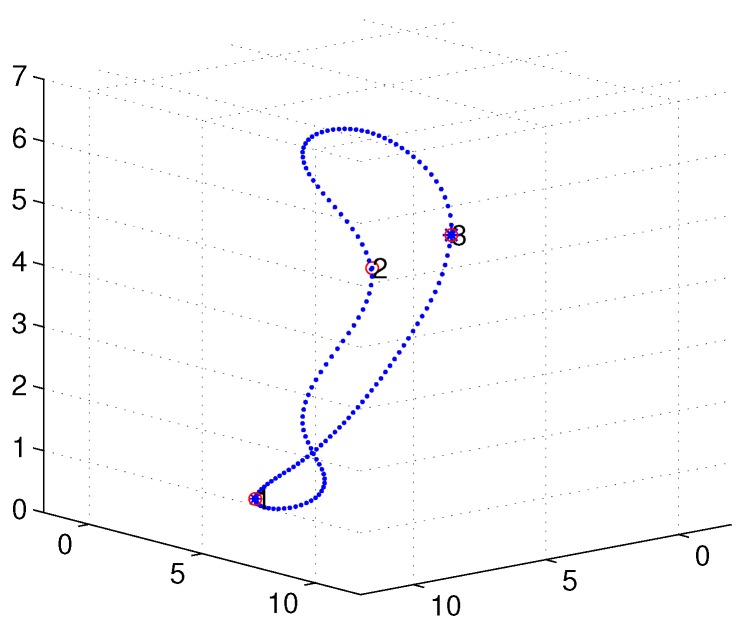
Three-dimensional (3D) Bezier interpolation results with n=3 nodes.

A simulation was set up with *n* sensor nodes randomly distributed in a cube of 10×10×10 unit${}^{3}$, with the AUV’s turning radius of r=1 unit for the Dubins path. The MATLAB 2016b simulator was used as the simulation tool. Figure 8 depicts an initial deployment snapshot of our simulation scenario when n=19, and Figure 9 shows the 3D interpolation results with only n=3 sensor nodes. It is intuitively clear that this simple curve derived by our proposed algorithm is continuous and smooth.

**Figure 10:**
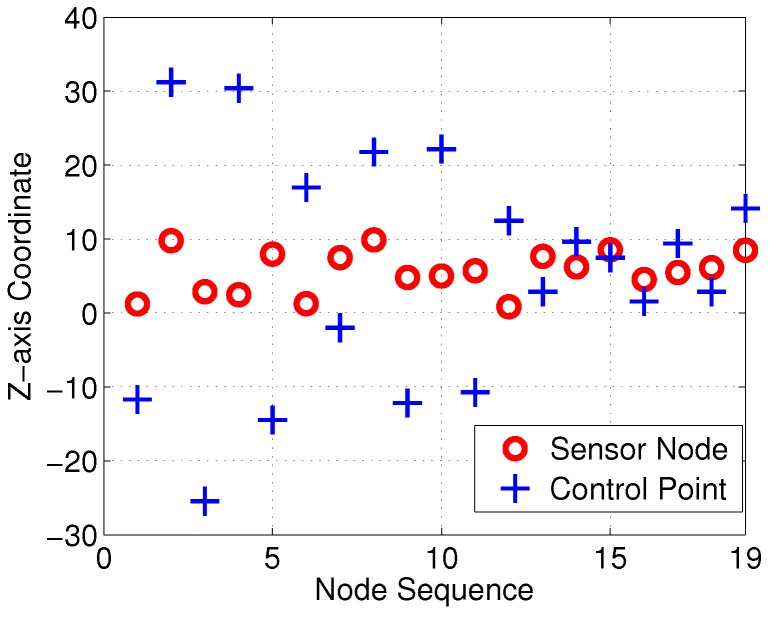
Sensor nodes and control points of Bezier curves.

**Figure 11:**
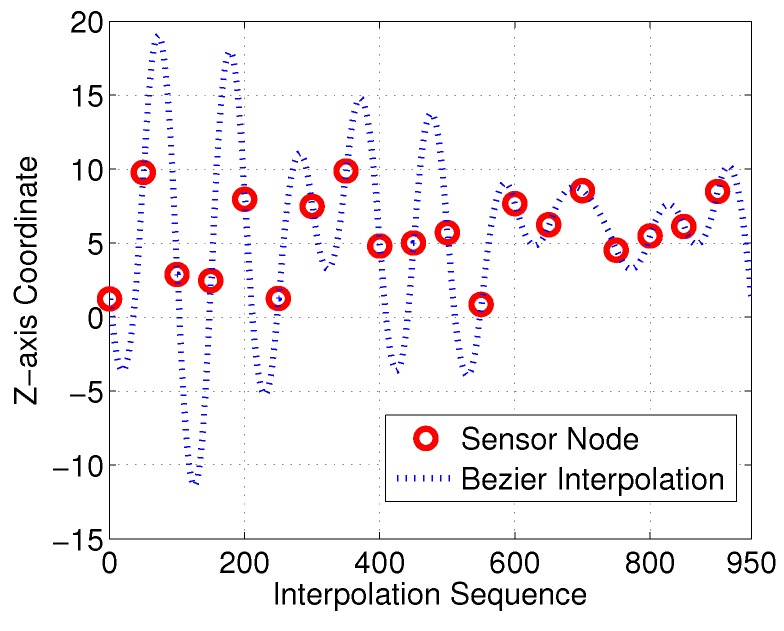
Bezier interpolation between sensor nodes in *Z*-axis.

With the proposed interpolation method of Section 3, the desired control points and interpolation results for the Bezier curves in the *Z*-axis are as illustrated in Figure 10 and Figure 11, respectively. Control points generated with Equation $M=A^{-1}S$ should satisfy the $3D-G^{1}$ continuous characteristic in the *Z*-axis.

**Figure 12:**
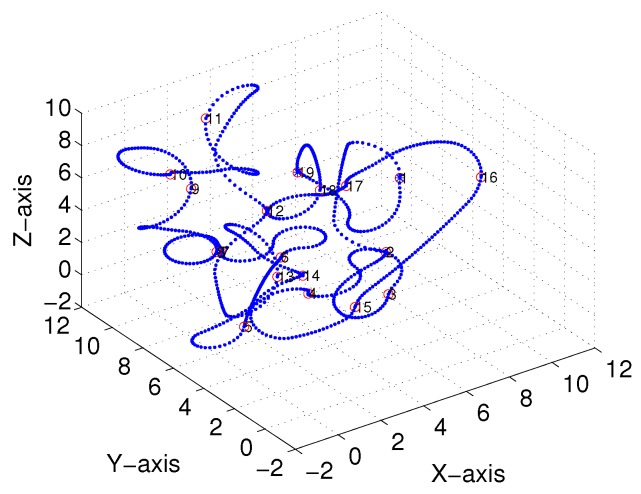
Linear-interpolation-based three-dimensional (3D) Dubins curves (case 1).

**Figure 13:**
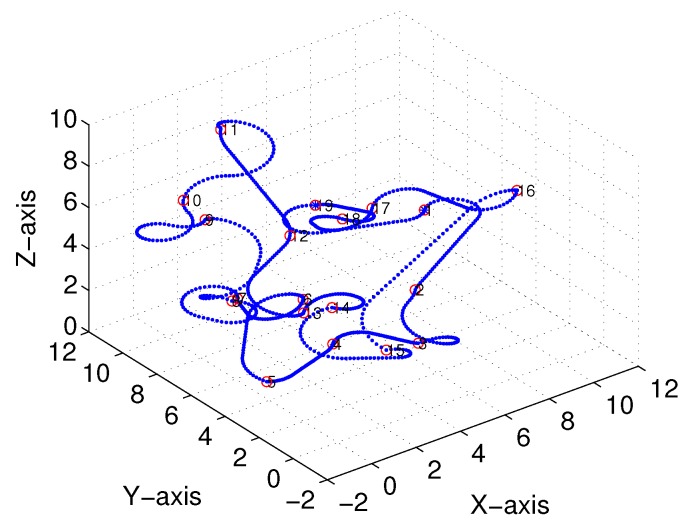
Bezier interpolation based three-dimensional (3D) Dubins curves (case 1).

**Figure 14:**
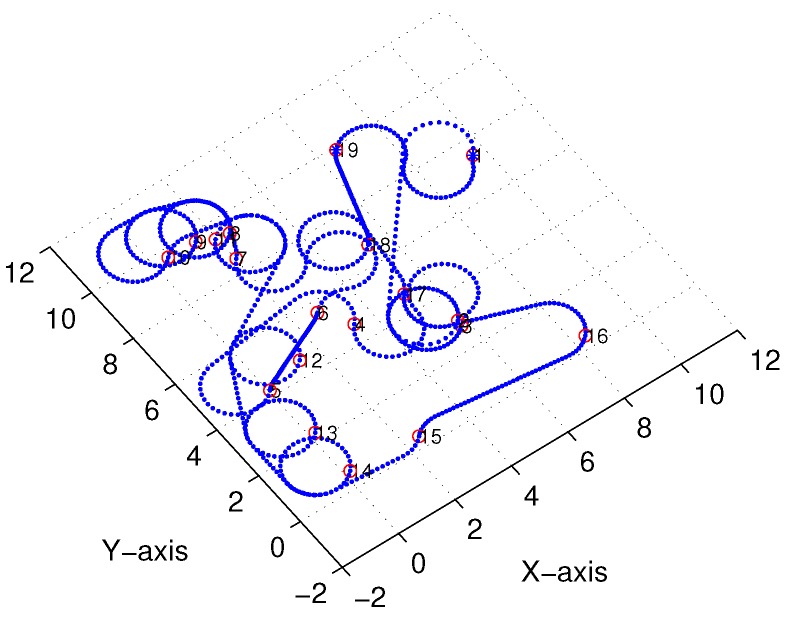
*X*–*Y* projection of linear-interpolation three-dimensional (3D) Dubins curves (case 1).

**Figure 15:**
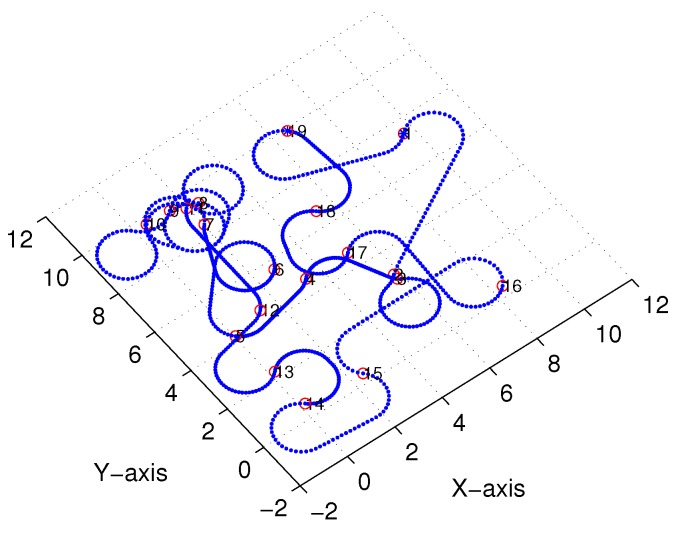
*X*–*Y* projection of Bezier interpolation three-dimensional (3D) Dubins curves (case 1).

Subsequently, following the methods proposed in [22,23] and Section 3, we obtained comparable results derived from the linear-interpolation-based 3D Dubins curves and Bezier interpolation based 3D Dubins curves, respectively. Figure 12 and Figure 13 illustrate the final trajectory of the linear-interpolation 3D Dubins algorithm and Bezier interpolation 3D Dubins algorithm when $\phi =\frac{\lambda \pi }{4}$, and their projection curves in the *X*–*Y* plane are shown in Figure 14 and Figure 15, respectively. It can be shown that the desired 3D curves are both continuous and smooth in the *X*–*Y* plane.

**Figure 16:**
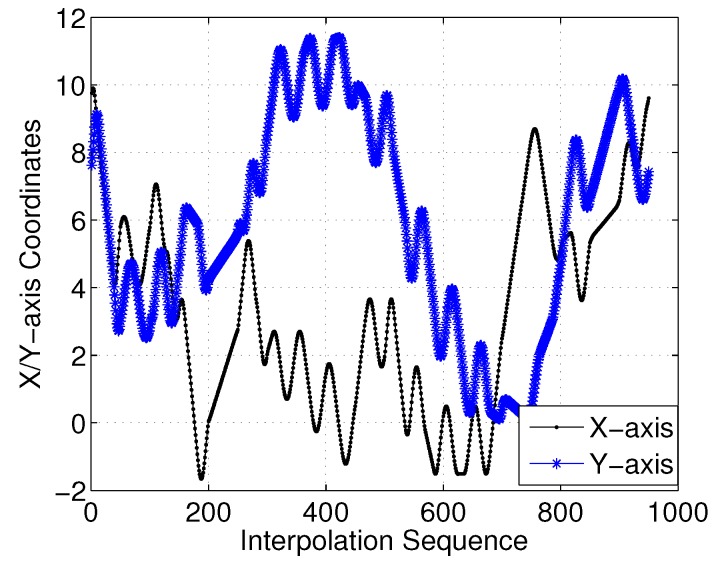
*X*/*Y*-axis of three-dimensional (3D) Dubins curves (case 1).

**Figure 17:**
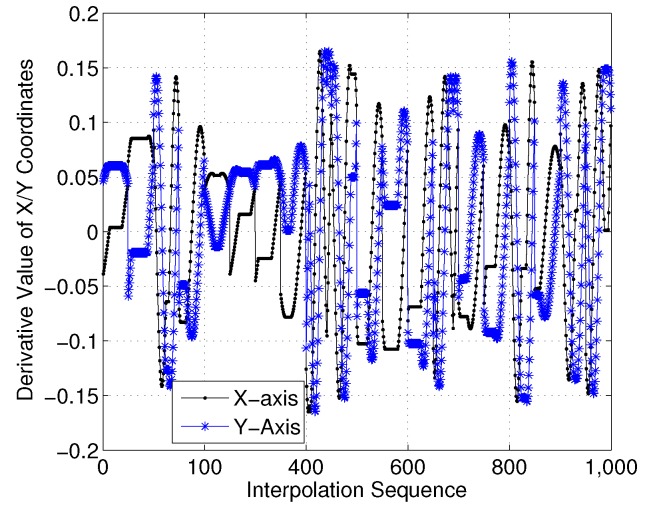
First-order derivatives of *X*/*Y*-axis coordinates (case 1).

To show this more clearly, Figure 16 gives the *X*- and *Y*-axis coordinates for each interpolation sequence. Moreover, Figure 17 presents the first-order derivatives of the *X*- and *Y*-axis coordinates. Clearly, the differential curve is continuous with the interpolation sequences.

**Figure 18:**
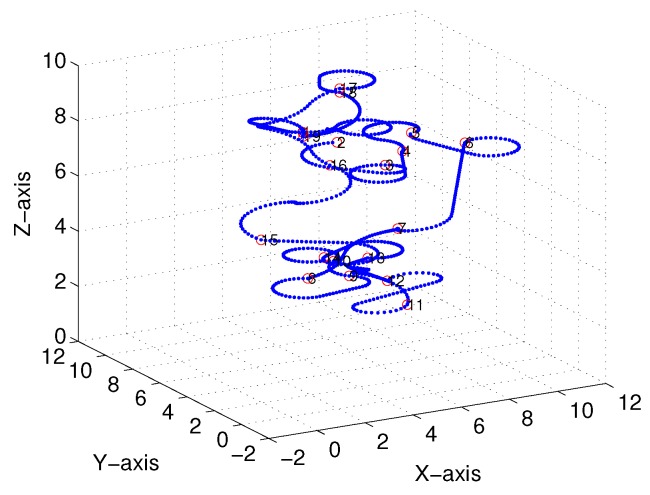
Linear-interpolation-based three-dimensional (3D) Dubins curves (case 2).

**Figure 19:**
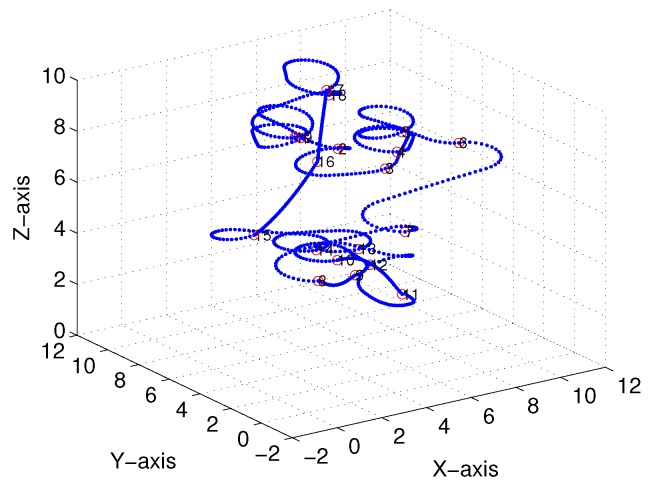
Bezier interpolation based three-dimensional (3D) Dubins curves (case 2).

**Figure 20:**
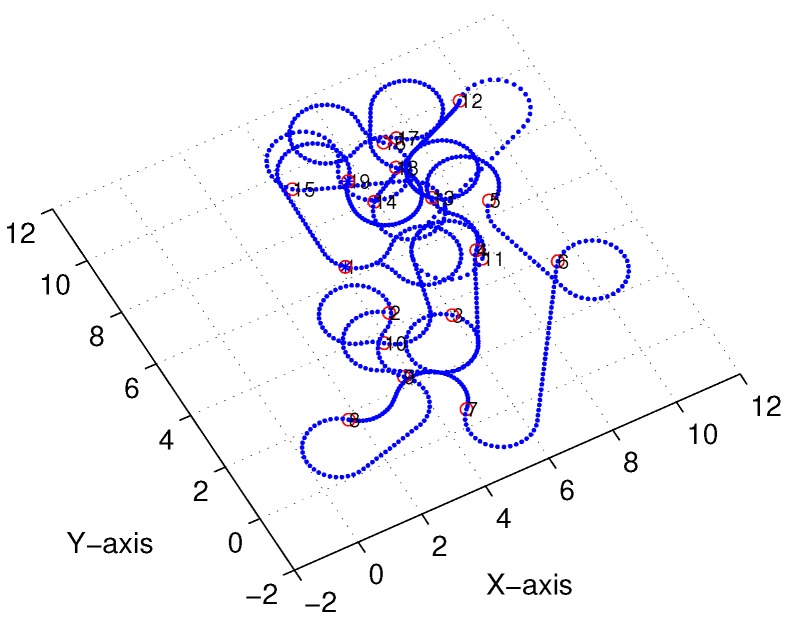
*X*–*Y* projection of linear-interpolation three-dimensional (3D) Dubins curves (case 2).

**Figure 21:**
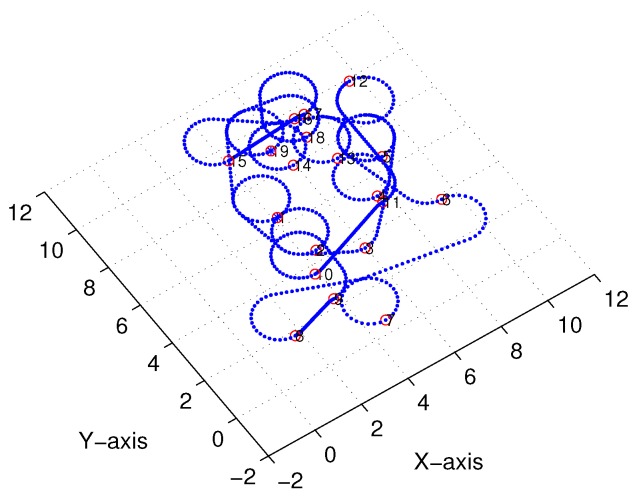
*X*–*Y* projection of Bezier interpolation three-dimensional (3D) Dubins curves (case 2).

Figure 18 and Figure 19 illustrate the final trajectory of the linear-interpolation 3D Dubins algorithm and Bezier interpolation 3D Dubins algorithm when $\phi =\frac{\lambda \pi }{6}$, and their projection curves in the *X*–*Y* plane are shown in Figure 20 and Figure 21, respectively. These curve interpolation results verify that 3D trajectories with linear interpolation and Bezier interpolation are both continuous and smooth in the *X*–*Y* plane with different azimuth heading sets (cases 1 and 2). However, more diverse azimuth headings indicate a more complicated iteration process. In the following simulations, we again used $\phi =\frac{\lambda \pi }{4}$ to reduce the computational complexity of the GA.

**Figure 22:**
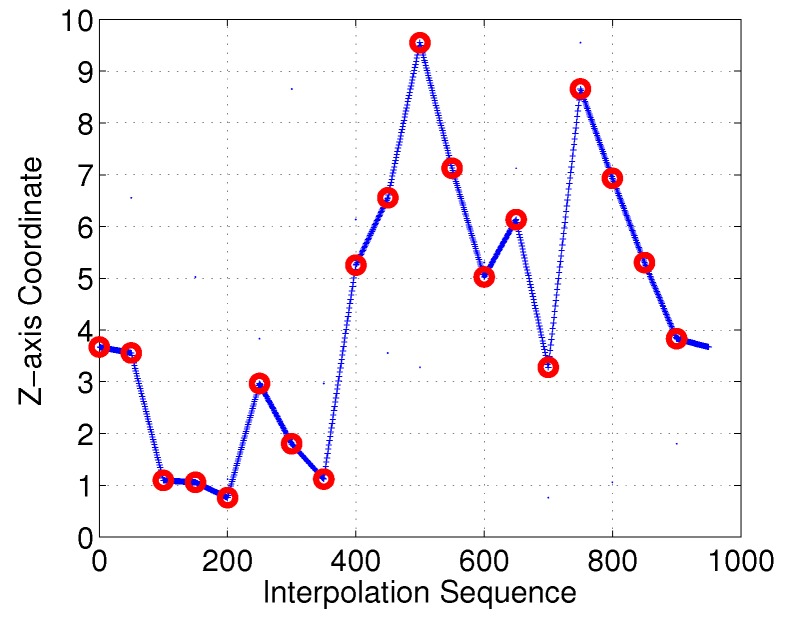
*Z*-axis of linear-interpolation three-dimensional (3D) Dubins curves (case 1).

**Figure 23:**
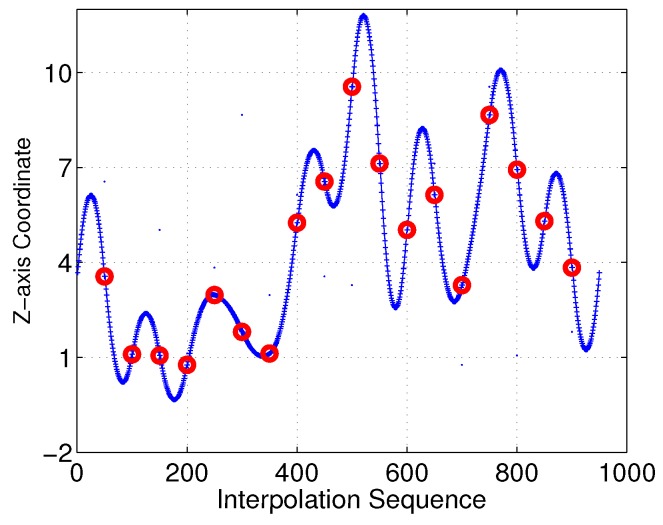
*Z*-axis of Bezier interpolation three-dimensional (3D) Dubins curves (case 1).

**Figure 24:**
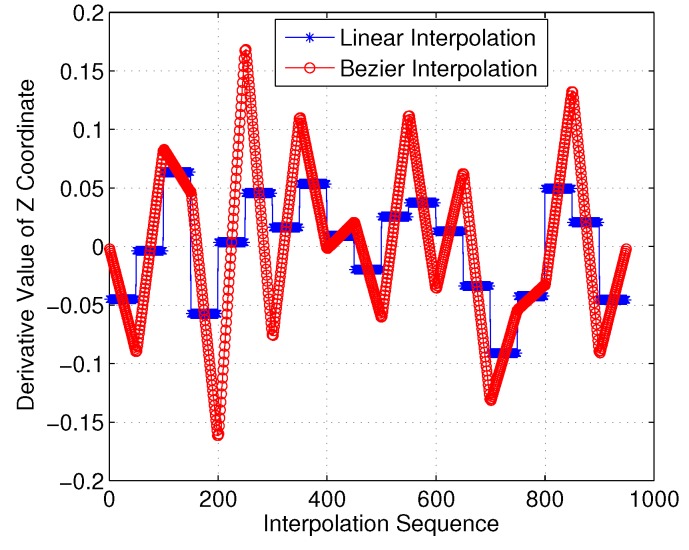
First-order derivative of *Z*-axis coordinate (case 1).

Moreover, we investigated the smoothness feature in the *Z*-axis. As shown in Figure 22 and Figure 23, it is clear that the linear-interpolation 3D Dubins method failed $G^{1}$ continuity in the *Z*-axis, while the Bezier interpolation 3D Dubins method satisfied $G^{1}$ continuity in both the *X*–*Y* plane and *Z*-axis. Furthermore, we compared the first-order derivatives of the *Z*-axis coordinate in Figure 24. It can be seen from the simulation results that the first-order derivative derived from Bezier interpolation was continuous, while that derived from linear interpolation was not continuous, thus proving that the proposed Bezier interpolation 3D Dubins curves belong to 3D smooth curves.

Finally, we computed the average total cruising lengths generated by the linear and Bezier interpolation based 3D Dubins algorithms with different numbers of underwater sensors. The average total cruising lengths of the two algorithms were derived from 100 Monte Carlo simulations. The comparison results in Table 1 verify that the Bezier interpolation based 3D Dubins curve planning algorithm had almost the same length compared to the linear-interpolation-based 3D Dubins curve planning algorithm whether for case 1 or 2. This means the Bezier interpolation method only needs a slightly higher path cost than the linear interpolation method. On the other hand, the energy consumption spent on the multi-hop acoustic transmission of underwater sensors is substantially reduced because sensor nodes only need to deliver sensory data to neighboring AUVs. As a result, the lifetime of entire USNs improves with the mobile data collection method.

## 5. Conclusions

This study investigated the smooth-path-planning-based mobile data collection problem for 3D USNs. The 3D Dubins curves based trajectory planning algorithm for MDG uses $G^{1}$ continuous Dubins curves in the *X*–*Y* plane and Bezier interpolation in the *Z*-axis to construct 3D smooth curves, so as to guarantee $3D-G^{1}$ continuity and therefore overcome AUVs’ nonholonomic constraints in 3D regions. Simulation results demonstrate that the proposed algorithm can support AUVs’ kinematic constraints and satisfy 3D smooth mobile data collection requirements with only a little extra cost. In our future works, sensor nodes’ passive drift by irregular sea currents will be considered. Moreover, obstacle avoidance will be considered in the process of mobile data collection path planning.

## Figures and Tables

**Table 1:** Average total cruising length of three-dimensional (3D) Dubins curves (unit). LI denotes linear-interpolation-based 3D Dubins path planning; BI denotes Bezier interpolation based 3D Dubins path planning.

Number of Sensors	n=3	n=7	n=11	n=15	n=19
LI algorithm (case 1)	20.3	51.1	80.5	118.7	136.2
BI algorithm (case 1)	22.5	55.6	90.0	127.3	159.7
LI algorithm (case 2)	23.1	55.3	80.3	117.7	135.9
BI algorithm (case 2)	27.5	57.2	85.3	125.3	152.2
